# A Combined Impact-Process Evaluation of a Program Promoting Active Transport to School: Understanding the Factors That Shaped Program Effectiveness

**DOI:** 10.1155/2013/816961

**Published:** 2013-03-31

**Authors:** S. Crawford, J. Garrard

**Affiliations:** ^1^Deakin University, 221 Burwood Highway, Burwood, VIC 3125, Australia; ^2^Parenting Research Centre, Level 5, 232 Victoria Parade, East Melbourne, VIC 3002, Australia

## Abstract

This mixed methods study was a comprehensive impact-process evaluation of the Ride2School program in metropolitan and regional areas in Victoria, Australia. The program aimed to promote transport to school for primary school children. Qualitative and quantitative data were collected at baseline and followup from two primary schools involved in the pilot phase of the program and two matched comparison schools, and a further 13 primary schools that participated in the implementation phase of the program. Classroom surveys, structured and unstructured observations, and interviews with Ride2School program staff were used to evaluate the pilot program. For the 13 schools in the second phase of the program, parents and students completed questionnaires at baseline (*N* = 889) and followup (*N* = 761). Based on the quantitative data, there was little evidence of an overall increase in active transport to school across participating schools, although impacts varied among individual schools. Qualitative data in the form of observations, interviews, and focus group discussions with students, school staff, and program staff provided insight into the reasons for variable program impacts. This paper highlights the benefits of undertaking a mixed methods approach to evaluating active transport to school programs that enables both measurement and understanding of program impacts.

## 1. Introduction

Over the last 30 years, rates of children walking and cycling to school in Australia have declined substantially [1–3]. This has been accompanied by increasing rates of children being driven to and from school [4]. Factors such as the built environment making active transport modes difficult, a culture of car dependence [5], concerns about personal safety and traffic danger among parents [6, 7], and an increase in parents' working hours [4, 8] have contributed to this trend. These trends at the social, cultural, environmental, and economic levels have been linked to a decrease in physical activity [9] and an increase in overweight and obesity among Australian children [10]. While Australian data are not available at the national level, comparative international data show an inverse association between active transport and childhood obesity [11]. There is also consistent evidence of multiple benefits (e.g., health, environment, and community liveability) of active transport for young people [12–15]. 

Until relatively recently, programs to promote physical activity among children focused on structured exercise programs, sport, and formal physical education in schools [16]. The promotion of active transport to school is relatively recent. Evidence reviews indicate that some, but not all, programs achieve small-to-moderate increases in rates of active transport to school [17–21]. Variable program impacts occur both between programs and for individual schools within multisite programs. 

Some more recent evaluations have been reported in Australia and New Zealand. The Central Sydney Walk to School Research Program, which comprised a randomised controlled trial involving 24 government primary schools in inner suburban Sydney, reported inconsistent evidence of an impact on students' walking trips to and from school. Parent-reported data showed an increase in students' walking trips, but student-reported data showed no significant changes [22]. Evaluation of the Brisbane Active School Travel (AST) program, delivered by the Brisbane City Council, reported a 24.8-percentage point increase in active travel mode share *to* school and an 18.1-percentage point increase in active travel mode share *from* school across the 13 primary schools that participated in the 2008 AST program. Walking trips to school increased by 19.1-percentage points (from 19.0% to 38.1%) and cycling trips by 3.1-percentage points (from 3.9% to 7.0%) [23]. In New Zealand, the Auckland Regional Transport Authority's 2007 School Travel Plan evaluation, based on “roll call” data collected from 35,153 students across 68 primary and secondary schools, reported a reduction in travel by “family car” of 3.4-percentage points and a 2.4-percentage point increase in walking and cycling [24].

Much of this evaluation literature is relatively recent, and there has been little systematic assessment of the reasons for variable program impacts. Implementing active transport to school initiatives and assessing their effectiveness in participating schools are important, but it is also important to examine the program and contextual factors that shape the effectiveness of interventions. This study aimed to increase the impact of the Ride2School program in a number of school communities. 

## 2. Methods

### 2.1. The Ride2School Program

The Ride2School program was conducted by Bicycle Network Victoria (a charitable institution that promotes and advocates for cycling throughout Victoria and Australia). From 2006 to 2010, the Ride2School program received $4 million funding from the Department for Victorian Communities, the Department of Human Services, the Victorian Health Promotion Foundation (VicHealth), Diabetes Australia-Victoria, and the Victorian Roads Authority (VicRoads). This study involved the first two years of the program (from 2006 to 2007).

The Ride2School program aimed at increasing the number of children using active transport to school, principally through behaviour change measures [25]. The program promoted walking and “wheeling” (cycling, scooter/skate) to school, but, in practice, placed greater emphasis on cycling. 

The Ride2School program was implemented in two main phases. In the initial pilot phase, three schools were offered a number of program activities (e.g., participation in Ride2School Day, mapping of safe routes to school, and classroom surveys to track active transport rates), as well as infrastructure improvements funded through the program (e.g., bicycle storage funding and, for one of the pilot schools, raised pedestrian crossings). The second phase of the program involved customised advice, support, and resources for 13 participating schools which self-selected to join the program. For approximately nine to 12 months in the pilot schools and six months in the program schools, participating schools were given guidance and hands-on support from one of two Ride2School Coordinators to assist with the planning and promotion of the program within the school. Program schools were offered similar program activities as in the pilot program, but without the infrastructure components. Key activities included three cycling and active transport events (Ride2School Day, Walk and Wheel-a-thon, and 500-kilometre Gold Medal Challenge); mapping quiet neighbourhood routes to school; a website; monthly email newsletter; and incentives (e.g., 1000 Bikes Student Leadership Rewards). Not all activities were implemented in all participating schools. Schools were able to choose the activities they would participate in based on the time, resources, and interest they had to commit to the program. There was no minimum number of activities that schools were strictly required to implement; however they were encouraged to participate in all activities. Phase 1 of the Ride2School program focused on grades 5 to 6 students, and this was expanded to include grade 4 students in Phase 2.

Deakin University was contracted to undertake an external evaluation of the Ride2School program, and the authors were members of the Deakin University evaluation team. The findings reported here were for two schools involved in the pilot program (“pilot” schools) and 13 schools involved in the second phase of the program (“program” schools). Two comparison schools were matched to the pilot schools. The schools were all government, coeducational primary schools located in metropolitan or regional areas of Victoria. Ethical approval for the study was obtained from Deakin University Human Research Ethics Committee and the Victorian Department of Education and Early Childhood Development.

### 2.2. Evaluation Design

This was a mixed methods study using a sequential explanatory design [26] to assess the implementation and effectiveness of the Ride2School program. The study was quantitatively driven with a quantitative core design and analytic methods and a qualitative sequential component to provide insight into the quantitative findings [26, 27]. This design allows for the measurement and interpretation of program impacts [26]. The impact evaluation component of the study used primarily quantitative data collection methods and analysis to measure program impacts. The process evaluation component of the study used primarily qualitative data collection methods and analysis to (i) describe and analyse key aspects of the program's implementation, and (ii) provide insights and understanding of program impacts. 

### 2.3. Phase 1: Pilot Schools

#### 2.3.1. Study Design

A controlled pre-post design was used to measure the impacts of the program on two of the three pilot schools that joined the program in July 2006. Two comparison schools were matched to the intervention schools based on school type (i.e., government coeducational schools), size, location (distance from pilot school), and sociodemographic characteristics. One pilot school and its comparison school were located in established, neighbouring suburbs, approximately 2 km apart and approximately 7 km from Melbourne's central business district (CBD) (inner suburban). The second pilot school and its comparison school were located 2 km apart in the same suburb, in a new, rapidly growing housing estate approximately 22 km from the CBD (outer suburban). Ensuring the matched schools were a short distance apart meant that the schools shared area-level similarities that impact on active transport such as street design features, population density, and access to public transport.

#### 2.3.2. Data Collection Measures and Procedures

The main data collection methods in the pilot phase of the study were (i) direct observation of active transport modes to school and counts of bicycles and scooters on school premises, (ii) classroom Hands Up! surveys, and (iii) qualitative interviews with Ride2School program staff. No Ride2School events (e.g., Ride2School Day) were conducted on the days when data were collected at the four schools.

Observational counts were conducted at each of the four schools at baseline (July 2006) and followup (August 2007). An observational checklist was developed based on a number of previously developed instruments [28, 29] and on program objectives. The checklist included the number of students using each active transport mode (walking, cycling, scooting, and skating), gender, time of day, and who the student was traveling with. The checklist also included a section for counts of the number of bicycles and scooters located in the school grounds. Observations were conducted at each of the schools between 8 am and 9:15 am (school started at 9 am). A team of observers were positioned near each of the school entrances to observe the travel modes of students arriving at the school and were positioned so they could distinguish between children walking from home and walking from a car (only the former were recorded as “walking”). The weather was similar for all data collection days (cool with no rain).

Hands Up! classroom surveys of grades 5 and 6 students at each of the pilot schools and comparison schools were used to collect information about students' modes of travel to school on the day of the survey and the previous four school days. The surveys were conducted by classroom teachers. The classroom survey was developed based on a previously developed instrument [29] and on program objectives. 

Qualitative data were collected with the Ride2School Coordinator working directly with the pilot schools program. The Coordinator participated in face-to-face or telephone interviews each month for the duration of the program and six months after the program, to provide information on how the program was progressing in each school, the supports and barriers to implementing the program in each school, and how barriers were being overcome.

#### 2.3.3. Data Analysis

For the observational data, proportions of students using active modes of travel to school (walking, cycling, and scooter/skating) were calculated based on school enrolment data. Differences between proportions in travel modes were calculated using *z*-ratio computations. Chi-square tests of significance were used to determine differences between demographic variables. Because three different data collection methods were piloted (each with advantages and limitations), the overall pattern of findings was used as a form of “data triangulation” to make judgments about program impacts.

### 2.4. Phase 2: Program Schools

#### 2.4.1. Study Design

Thirteen program schools participated in Phase 2 of the study which was an uncontrolled pre-post design. Attempts to recruit a sufficient number of matched comparison schools within the time constraints (i.e., before implementation of the program commenced) were unsuccessful. Three schools invited to participate as comparison schools subsequently joined the Ride2School program as program schools, forming part of the evaluation. Schools that declined to participate cited lack of time and frequent requests for other data collection in schools as reasons for not wishing to participate. Therefore, comparison schools were not included in this phase of the study. Of the 13 program schools, nine schools were located in regional areas of Victoria, and four were located in metropolitan Melbourne. Government primary schools in Victoria with an interest in promoting active transport were eligible to apply to be involved in the program. Being located in an area of disadvantage was also beneficial to schools applying, but not compulsory, and the 13 schools included schools from both disadvantaged and more advantaged areas. 

#### 2.4.2. Data Collection Methods and Procedures

The main data collection methods in this phase of the study were: (i) parent and student surveys, (ii) interviews with principals and Ride2School Coordinators, and (iii) focus group discussions with teachers and students. 

Parents of all grades 4, 5, and 6 students were invited, via an information package sent home with students, to participate in a written survey about their child's travel to school behaviour and parents' attitudes to school travel modes. All students in grades 4, 5, and 6 were invited to participate in a written survey of how they traveled to and from school on the day of the survey and for the previous four days and about their attitudes to different ways of traveling to and from school. Written parental consent was required for student participation. The student surveys were administered by teachers and were completed in class by students who returned consent forms from their parents or guardians and who agreed to participate in the survey.

Parent and student surveys were conducted before (March 2007) and after (November 2007) the implementation of the Ride2School program in the program schools. The program was implemented over two school terms from March to September (from Autumn to Spring). 

Because student and parent surveys were anonymous, parent and student data were not matched and were analysed separately. For similar reasons and also to maximize response rates, study participants (students and parents) were not matched at baseline and followup.

Across the 13 schools, participants in individual interviews and focus group discussions included 11 school principals, 21 teachers, 70 students (across grades 4, 5, and 6), and two Ride2School Coordinators. School principals were invited directly to participate in an interview, and they were asked to nominate approximately three teachers who might be interested in participating in a focus group. The teachers needed to have had some involvement with the program (e.g., the “cycling champion” or a grade 4, 5, or 6 teacher) to participate. Principals, teachers, and program staff provided written consent to participate in an interview or focus group, and parents provided written consent for their child to participate in a focus group. 

Data collection instruments were developed based on program and evaluation objectives. Student and parent surveys were pilot tested with 10 grade 4–6 students and parents, and minor amendments were made. Each of the interviews and focus groups was semistructured, with an interview schedule that outlined the main topics and issues to be covered in the interview or focus group [30].

Individual qualitative interviews were conducted with principals via telephone to provide insight into the reasons for the school participating in the Ride2School program, their views on how the program was implemented in their school, and suggestions on how the program could be improved. Focus group discussions were conducted with teachers to determine their observations of the program, the supports and barriers to implementing the program, and how they thought the program was received by students. Focus group discussions were conducted with students to gain an understanding of their attitudes to active transport and general feedback on the program. The two Ride2School Coordinators who worked directly with the schools participated in monthly, face-to-face or telephone interviews and provided information on how the program was progressing in schools, the supports and barriers to implementing the program in each school, and how barriers were being overcome.

#### 2.4.3. Data Analysis

Analysis of quantitative data was conducted with SPSS version 14.0.1 and Stata version 10.1. Descriptive statistics were generated for all quantitative study variables. Differences between proportions in travel modes were calculated using *z*-ratio computations. Chi-square tests of significance were used to determine differences between demographic variables, and independent *t*-tests were used to compare the differences in means for child age. In Phase 2 of the study, logistic regression models were computed to examine the impact of the Ride2School program, including adjustment for possible correlates of active transport to school. Details are included in the results section.

Analysis of the qualitative data from the interviews and focus groups in each school involved a number of steps, as described by Green and colleagues [31]: immersion in the data, coding, creating categories and identifying key themes. Categories and themes were then compared for the 13 program schools, to identify patterns in the data across schools.

## 3. Results

### 3.1. Phase 1: Pilot Schools

#### 3.1.1. Quantitative Data

Based on the observational counts of students using active modes of travel to school in 2006, active transport was more common in the two inner suburban schools (pilot and comparison schools) (36.1% of trips) compared with the two outer suburban schools (pilot and comparison schools) (22.3%; *P* < 0.0002). Data for 2007 were similar.

In the inner suburban schools, observational data for all active transport modes combined showed an increase in rates of active transport from 2006 to 2007 in the pilot school (+7.6%; *P* = 0.033) and a decrease in the comparison school that was not statistically significant (−4.0%; *P* = 0.256) (Table 1). Although there were small increases in rates of active transport to school in both of the outer suburban schools from 2006 to 2007, the increase was significant for the comparison school only (+5.2%; *P* = 0.025). 

Active transport rates based on the classroom Hands Up! surveys of grades 5 and 6 students were generally higher than for the observational counts, and this is likely due to the older sample, as older children are more likely to use active transport to school [13, 32, 33]. For the inner suburban schools, Hands Up! survey data showed an increase in walking and cycling trips to school for the school week in the pilot school (+8.8%; *P* ≤ 0.0002) and no significant change in these trips to school in the comparison school (−4.3%; *P* = 0.200) from 2006 to 2007 (Table 1). In both of these schools, bicycle counts in the school grounds identified more bicycles in 2007 than in 2006, but in neither school was the change in the number of bicycles significant (Table 1). 

In both of the outer suburban schools, classroom Hands Up! surveys of grades 5 and 6 students showed no significant changes in walking and cycling to school from 2006 to 2007 (see Table 1). In the pilot school, the number of bicycles counted in the school grounds declined between 2006 and 2007 (−4; *P* = 0.015) (see Table 1).

Overall, based on data from the three sources, there is reasonably consistent evidence of an increase in rates of active transport to school from 2006 to 2007 in the inner suburban pilot school, relative to the comparison school. However, in the outer suburban schools, the picture is not as consistent. The observational counts showed small nonsignificant increases in rates of active transport in both the pilot and comparison schools, while the classroom Hands Up! surveys showed small nonsignificant decreases in active transport, and bicycle counts showed a decrease in the number of bicycles in the school grounds at the pilot school, indicating less cycling to school. The three data sources therefore provide little evidence of a program impact in the outer suburban pilot school.

#### 3.1.2. Qualitative Data

Qualitative data collected as part of the process evaluation can assist in explaining the impact evaluation data described above. Environmental characteristics of the study areas provide information about school contexts and implementation factors, based on qualitative data collected from the Ride2School Coordinator, and provide insight into the program's implementation in the pilot schools.

Both of the inner suburban schools (pilot and comparison schools) were in neighbourhoods of relatively high dwelling density and street connectivity, environmental characteristics that have been associated with walking and cycling for transport [34–38]. Both schools were serviced by train stations within 500 meters.

The two outer suburban schools (pilot and comparison) were in areas of lower dwelling density with streets of lower connectivity, when compared with the inner suburban areas. Personal observations showed that the outer suburban pilot school was not located within easy or safe access of much of the residential areas. To access the school, students needed to travel on a busy arterial road, cross the creek over a bridge with a narrow shared pedestrian/cyclist footpath, cross the main road at the school crossing and travel past heavy machinery on a large commercial building site next to the school. Accessing the outer suburban comparison school appeared to be easier and safer, with housing located closer to the school and wider footpaths for pedestrians and cyclists, although streets were of fairly low connectivity. There was a school bus traveling to each of the schools.

Factors that both enabled and constrained the program's implementation in the two pilot schools occurred at the program level, school community level, and the environmental level. These factors varied for the two program schools and were identified through semistructured interviews with the Ride2School Coordinator. 

*Supports for Program Implementation*. The Ride2School Coordinator undertook more intensive work with the pilot school in the inner suburban area than in the outer suburban area. He was more “hands-on” with this school, and therefore the teacher “cycling champion” was not expected to do too much work. There were a number of challenges (e.g., developmental stage of the school, priorities, and environmental factors) in working with the outer suburban pilot school.

Infrastructure improvements were made in the inner suburban pilot school as part of the program, which were not made to the same extent in the outer suburban school. Both schools received $4000 funding to improve their bike storage; however in the inner suburban pilot school a bike shed was erected quickly, and, in the outer suburban school, a temporary bike shed was provided for the first year until a more permanent shed could be built. In addition, two raised pedestrian crossings were built next to the inner suburban school, as part of the program, which were not provided to the outer suburban school.

As much as the environmental characteristics of the school areas were important, further qualitative data suggest that the culture in inner suburban communities (e.g., higher levels of walking and cycling and less car-dependence, in the general community) could make it easier to increase rates of active transport in these areas, as well as working with middle-smaller sized schools, such as the inner suburban pilot school. The school community was important in the implementation and acceptance of the program. At the inner suburban pilot school, the evidence suggested an enthusiastic and committed school community that made good use of resources available through the Ride2School program. In contrast, the outer suburban pilot school was in the process of establishing itself in a rapidly developing area and faced a number of competing priorities requiring considerable attention, resources, and staff time.

In summary, the program appeared to have greater impact in the inner suburban pilot school than in the outer suburban pilot school. Qualitative data suggest that the program was easier to implement and promote within a school that was smaller, more established, with a culture that was accepting and enthusiastic about active transport, in an area of higher density and lower car use, with greater use of infrastructure improvements and a more “hands-on” approach from the program Coordinator. 

### 3.2. Phase 2: Program Schools

#### 3.2.1. Quantitative Data

*Parent Surveys*. Surveys were completed by 410 parents at baseline (28.7% response rate), and 358 parents at followup (25.1% response rate). The majority of parents completing the surveys were female (89.0%, *P* = 0.920), and more than half of parents were in the 40–49 years age range (Table 2). Approximately two-thirds of respondents had completed some qualification since leaving school. There was a significant difference for parents' educational attainment at baseline and followup (*P* = 0.042), although approximately 3% of respondents did not answer this question at both time points. 

Baseline and follow-up parent surveys included questions about children's modes of travel to and from school. Post program, there was a small nonsignificant decrease in the proportion of trips to and from school by car for five consecutive weekdays (from 46.3% to 44.2%, *P* = 0.076) and a corresponding small increase in active trips that was not significant (from 47.9% to 49.6%, *P* = 0.125), due to a significant increase in cycling (from 13.9% to 15.9%, *P* = 0.015) (Table 3). 

Logistic regression analysis was used to assess whether levels of students' active transport to school, as reported by parents, were significantly different at followup relative to baseline. In order to detect possibly small changes in active/inactive trips to and from school among students, a binary outcome variable was created (no active trips in the last five school days = 0, 1–10 active trips in the last five school days = 1).

The analysis was conducted in three stages. First, the crude association between active transport and the intervention (baseline = 0, followup = 1) was examined. At followup, the proportion of students using 1–10 trips to and from school by active transport was 5.9% higher than at baseline (62.9% at baseline, 68.8% at followup). A chi-square test for difference in proportions and a simple logistic regression showed that this difference was not statistically significant (*P* = 0.087). 

Second, regression analysis was conducted accounting for clustering by school. Schools with parent and student participant numbers less than 20 at either baseline or followup were combined for clustering, based on school location, environmental context, and how the program was implemented and supported by schools. This resulted in eight school clusters. After accounting for clustering by school, no association was found between the intervention and students using active transport to or from school at least once per week (adjusted OR = 1.30, 95% CI = 0.95–1.79, *P* = 0.107).

Third, to account for potential confounding factors, a number of predictor variables were added to the model (Table 4). Significant results (using chi-square test of difference in proportions) for associations with children's use of active transport to or from school were simultaneously entered into the model in one block, to adjust for covariates that may affect the relationship between the outcome (active trips) and the data collection period (intervention) (Table 5). After accounting for clustering by school and adjusting for predictor variables (school location (regional or metropolitan), distance to school, number of cars per adult in household, child grade, how often spouse/partner cycles, and possibility of regular walking/cycling to school), a statistically significant increase in active transport post intervention was detected (adjusted OR = 1.67, 95% CI = 1.04–2.68, *P* = 0.035). 

*Student Survey. *Surveys were completed by 479 students at baseline (33.6% response rate) and 403 students at followup (28.3% response rate). Student respondent characteristics are shown in Table 6. There were broadly similar proportions of students in grades 4, 5, and 6. The average age of students was significantly higher at followup than baseline (11 years and 10 years, respectively, *P* < 0.0001), consistent with the timing of the baseline and followup surveys (predominantly in March 2007 and November 2007).

Student-reported data were broadly similar to parents' reports on students' travel modes to and from school, including those using active and inactive modes (Table 7). However, post program, there was a small nonsignificant increase in the proportion of trips to and from school by car for five consecutive weekdays (from 42.9% to 43.1%, *P* = 0.920), and a significant decrease in active trips (from 51.1% to 48.7%, *P* = 0.029), mostly from trips by scooter/skate (from 7.2% to 4.9%, *P* ≤ 0.0002). This differs from parent-reported data, which showed a small, though not significant, increase in active transport.

As with the parent data, analysis of the student data was undertaken in three stages using logistic regression to assess whether levels of students' active transport to school, as reported by students, were different at followup relative to baseline. At followup, the proportion of students using active transport to and from school at least once per week (1–10 trips) was 5.0% lower than at baseline (70.8% at baseline, 65.8% at followup). A chi-square test for difference in proportions and a simple logistic regression showed that this difference was not statistically significant (*P* = 0.109). After taking into account clustering by school, no statistically significant difference was detected between baseline and followup for students using active transport to or from school at least once per week (*P* = 0.409). Potential predictor variables were then added into the model to account for possible confounders. After accounting for clustering by schools and adjusting for the only significant predictor variable (school location) (Table 8), no statistically significant difference in active transport after the intervention was detected (adjusted OR = 0.80, 95% CI = 0.46–1.38, *P* = 0.421). 

In summary, for the 13 schools that participated in Phase 2 of the Ride2School program, there was inconsistent evidence of a postprogram change in grades 4, 5, and 6 students' rates of active transport to school. Parent-reported data showed a significant increase in the proportion of students using active transport to school at least once a week after adjusting for potential confounding factors, but student-reported data indicated no statistically significant change.

Similar to Phase 1 of the Ride2School program, impacts varied across the 13 schools that participated in Phase 2 of the program. Qualitative data collected as part of the process evaluation can assist in providing some insights into variable program impacts. School communities are complex socioenvironmental entities that interact with externally initiated programs such as the Ride2School program in complex ways. As noted in the “Realistic Evaluation” approach to program evaluation, in addition to assessing the net impact of interventions in multisite programs, it is also important to explore “What works for whom under what circumstances?” [39].

#### 3.2.2. Qualitative Data

The qualitative data were initially organised in the form of brief case studies of individual program schools. Quantitative data from parent and student surveys were added to the qualitative data to form an overall picture of the Ride2School program in each school, covering school context, program impacts, and program implementation. 

The motivations, supports, and barriers to implementing and promoting the Ride2School program differed for each of the 13 program schools. A summary of these factors, across the 13 program schools, is as follows. 

*Variation in Program Implementation. *Schools had different levels of interest, commitment, support, and resources to implement the program and to implement it well. Therefore, the program was implemented to varying degrees at each school. However, the number of activities schools participated in did not appear to predict changes in active transport rates. Most were one-off activities such as the Ride2School Day (a yearly event) that were relatively easy to organize and implement, but appeared not to result in sustained, schoolwide change.

Schools had different expectations of the Ride2School program in terms of how the program would be implemented in their school and the assistance that would be provided by the Ride2School Coordinators. In some cases, these expectations were at odds with both the expectations of the Coordinators and the services and resources provided through the program. This resulted in schools' expectations often not being met. The Ride2School Coordinators often had different perceptions (from those of teachers and principals) about what motivated schools to participate in the program; what some of the supports and barriers were to promoting the program; and generally how well the program was implemented. The three schools that appeared to experience increased rates of active transport to school post program were schools that the Coordinators found difficult to work with. According to Ride2School Coordinators, program impacts did not appear to reflect program implementation. In contrast, increased levels of active transport to school appeared to be associated more with highly motivated school communities, including parents, situated in supportive physical and sociocultural environments, who drew on the resources provided through the Ride2School program to further support what they were already doing. 

*School Setting and Surrounding Environment. *In terms of the physical and social environments associated with the schools, there was a clear distinction between schools located in regional areas and those in metropolitan areas. Many of the regional schools were constrained by being located near busy roads and long distances to travel to school, resulting in travel by car or school bus. On the other hand, metropolitan schools were mainly in inner suburban areas, with infrastructure that is more supportive of active transport (e.g., walking/cycling paths, high density, and street connectivity).

Many schools referred to the “car culture” in the school community as a barrier to promoting travel ehavior change. This appeared to have been less of an issue in metropolitan schools, who identified a local culture of environmental concern and/or sustainable practices (including active transport) as supports for the program.

All of the regional schools were in disadvantaged areas and appeared to experience more barriers to implement the Ride2School program than metropolitan schools. For example, regional schools appeared to have other important issues to deal with, which meant that “add-on” programs like the Ride2School program received less attention than other key priority issues. Several of these schools appeared to require greater levels of support and resources to implement the program than the metropolitan schools. 

*Program Impacts on Individual Program Schools. *There was some evidence of a program impact in three program schools (sample sizes precluded testing for statistical significance), one in a metropolitan area and two in regional areas. Qualitative data suggest that the combination of secure bicycle storage facilities, the promotion of all active transport modes (walking, cycling, and skating) equally by committed and energetic school staff, and a school and/or local community culture of active transport (including parents accompanying children to school by active transport) may have contributed to increased rates of active transport to school in these schools. 

## 4. Discussion

Evaluation of the pilot phase of the Ride2School program, which involved an inner suburban primary school and an outer suburban primary school, indicated an increase in active transport to school in the inner suburban school, but not in the outer suburban school. Data from qualitative interviews and an examination of the socioenvironmental characteristics of the two school communities suggest that program, school community, and socioenvironmental factors contributed to the differing outcomes. 

The inner suburban school was a relatively small (approximately 360 students from preparatory to grade 6), established school with a high level of interest in, and commitment to, increasing active transport to school. The school made good use of the resources available through the Ride2School program, including infrastructure improvements in and around the school. The school was also located in an area with higher rates of walking and cycling among the general population than in the greater Melbourne metropolitan area [40]. Children are more likely to use active transport to school in areas where active transport is more prevalent in the wider community [41]. 

The outer suburban school, on the other hand, was located in a rapidly developing area with relatively poor access by foot or bicycle to the school from surrounding residential areas. Trip distances also appeared to be greater than for the inner suburban school. The area has poor public transport, low levels of active transport, and high levels of car use [40]. As a new school undergoing rapid expansion (the number of enrolled students increased from 507 in 2006 to 846 in 2007), the school faced a number of challenges which meant that, while there was interest in promoting active transport to school, other issues had higher priority.

Findings from this pilot phase of the Ride2School program provide some support for the “Realistic Evaluation” approach to program evaluation which emphasizes the importance of not only measuring the aggregate effect of a multisite intervention, but also gaining a more nuanced understanding of “What (program factors) works for whom (population factors) under what circumstances (socioenvironmental factors)?” [39, 42]. The Community-Based Social Marketing model (on which the Ride2School program was loosely based) also highlights the importance of understanding how the supports and barriers to active transport vary across settings and population segments [43]. In terms of increasing active transport to school in a school setting with substantial barriers to active transport, it is likely that more intensive, targeted, and sustained behaviour change measures are required, in addition to environmental measures designed to make active transport to school a realistic, safe, appealing, and convenient alternative to car travel. This is consistent with recommendations from the evaluation of the TravelSmart Schools program in New South Wales, which advised that future programs to promote active transport to school should be implemented with greater intensity at fewer schools, over a longer period of time and for at least two years [44]. 

Evaluation of Phase 2 of the Ride2School program found inconsistent evidence of program impacts in the 13 participating schools. Parent-reported travel data indicated an increase in the proportion of grades 4, 5, and 6 students using active transport to school at least once a week, but student-reported data found no significant change. Differences between parent-reported and student-reported travel to school behaviour were also found in the evaluation of a program promoting walking to school in Sydney, where parent-reported data indicated a program impact, but student-reported data showed no significant program impact [22]. Given that research literature on the reliability of parent-proxy and student-reported data on travel to school shows both sources to be reliable [45–47], there is no consistent evidence that one source is preferable to the other for children in this age group. This study therefore concludes that there was inconsistent evidence of an overall program impact in the 13 schools that participated in the second phase of the Ride2School program, with differences between parent and student data likely due to a range of methodological issues discussed in more detail below.

Sample size limitations precluded a quantitative assessment of program impacts in individual schools, but there were some indications of a small-to-modest program impact in one metropolitan and two regional schools. These apparently successful schools did not appear to differ markedly or consistently from less successful schools in terms of extent or quality of program implementation or the key socioenvironmental correlates of active transport to school [41]. However, it is of interest to note that the Ride2School Coordinators described them as operating fairly independently of the Ride2School program, suggesting a high degree of commitment to active transport to school and the ability to draw on multiple resources (including, but not limited to, the Ride2School program) to foster active transport to school. They also appeared to have more widespread school and community support for active transport to school, rather than being dependent on one or two teachers (often keen cyclists) to promote the program. Previous research has found that programs using a community participation and/or development approach have been successful in producing an increase in active transport to school internationally [48, 49] and in Australia [50, 51]. 

Other factors that may have limited the success of the Ride2School program include the stronger focus on cycling to school than on walking to school. Other active school travel programs in Australia have found it easier to increase rates of walking to school than cycling to school [23], possibly due to generally poor cycling infrastructure and adverse traffic conditions in Australia. 

In the early years of the program (the subject of this study) the focus of the program tended to be on high profile one-off events and activities designed to create a profile for the program and, in the words of one interviewee, “get some runs on the board.” More recently, the program has incorporated capacity building within schools (e.g., professional development opportunities for teachers) and assisted schools to conduct more regular active transport activities (e.g., Walking Wheeling Wednesdays) [52].

The program focused on working directly with students and teachers, and there was limited active participation from parents. Parental involvement in travel behaviour change is crucial because parents are the principal decision-makers for how children of primary school age travel to school [53]. While parental participation in school programs is not always easy, it is important that parents be involved in identifying the benefits and barriers to active transport and car travel and suggesting strategies to address these benefits and barriers [54]. 

Apart from providing the inner suburban primary school in the pilot phase of the program with a school crossing and secure bicycle storage, the Ride2School program was predominantly a behaviour change program. In the program schools, it did not involve any substantial infrastructure changes in most schools (two schools received funding for bike storage improvements through Ride2School). Travel behaviour change programs such as those promoting active transport to school are often seen as a low-cost approach to achieving a mode shift from car travel to active transport [55–57]. However, there is some debate in the literature regarding whether substantial, sustained levels of active transport to school can be achieved in the absence of an integrated package of measures including improved walking and cycling infrastructure and traffic calming measures [41, 58–62]. Given the impact of the physical environment on active transport [33, 34, 58, 63], it meant that schools participating in this program with existing infrastructure that supported active transport, such as bicycle paths and secure bicycle storage, might have been better placed to successfully implement behaviour change measures.

Finally, the relatively short time period for program implementation (approximately six months) may have been too short to change school travel behaviour. Change in mode share from inactive to active trips to and from school can be difficult to achieve in the short term [18, 24]. 

In common with many studies of the impacts of active school travel interventions [18], this study has several methodological limitations. As an external evaluation working within the time constraints of the program, recruiting adequate and sufficient comparison groups into the evaluation prior to baseline data collection was difficult, therefore placing limitations on the study design. These issues reflect the difficulties of working within a “real world” program, rather than a controlled community intervention trial [64].

The pilot and program schools self-selected to participate in the Ride2School program, and many of the 13 program schools had relatively high rates of active transport to school at baseline (particularly for cycling) compared with Victorian state-level data [65], suggesting that schools with an existing interest in active transport to school may have self-selected into the program. Recruiting schools with a preexisting interest in active school travel might be advantageous in terms of working with motivated schools; however, it might also make further increases in active transport difficult as active transport is not a feasible or appealing option for all parents and students (e.g., those who live too far from school, have no safe route to school, or simply prefer to drive). 

In Phase 2 of the study, time constraints prevented testing of the validity and reliability of the data collection measures. Further, parent and student survey response rates were low (approximately 30%). While this is not unusual when active parental consent is required for research conducted in schools (as in this study) [66, 67], the low response rates may have led to nonresponse bias. This might have resulted in an overestimate of rates of active transport to school, though this bias is likely to be similar at baseline and followup. This study relied on classroom teachers to distribute surveys and consent forms to students in a timely manner, and their commitment to this varied. This highlights the difficulties of conducting research in schools that require active participation from school staff and parents. 

The study may also have been underpowered (i.e., too few schools and parent and student respondents) to detect the small changes in travel mode share reported in evaluations of similar programs in Australia and internationally [18]. As noted by Sullivan and Percy (2008) large sample sizes are required to rigorously measure these small changes. Although some evaluations of active school travel programs have reported substantial increases in active transport, those that have used more rigorous evaluation designs have generally reported smaller impacts (most commonly in the range of 0–4%) [18, 22, 24]. The observational methods (counts of students using active transport modes and bicycles on school grounds) used in the evaluation of the pilot program could have been used to add strength to the data from the surveys (overcoming biases associated with nonresponse, social desirability, and inaccurate responses). However, these observational counts are very resource-intensive and were not practical, given the resource constraints of the study, to be conducted at all schools, particularly schools in regional areas. 

## 5. Conclusion

Based on the substantial health, environmental, transport, and community benefits of active transport to school, the promotion of active transport to school is potentially a worthwhile investment [68]. However, evidence for the effectiveness of programs aimed at increasing children's rates of active transport to school is inconsistent, with variable program impacts occurring both between programs and for individual schools within multisite programs [18, 22, 44]. 

This study also reported mixed evidence for the effectiveness of the Ride2School program. However, use of a mixed methods impact-process evaluation design enabled exploration of the impact of contextual factors on the effectiveness of the active transport intervention in schools. In this study, relatively intensive support for active transport to school, in an inner suburban primary school that was located in a supportive environment for active transport and had a strong commitment to promoting active transport, appeared to be effective in increasing active transport to school. Less intensive support and resourcing of a larger number of primary schools (13) in inner suburban areas of Melbourne and regional areas were less effective in increasing active transport to school, though a small number of schools achieved small-to-modest increases in active transport to school. 

Important questions for future evaluation research into programs promoting active transport to school include gaining a better understanding of the program, school, population, and contextual factors that shape the effectiveness of interventions; the optimal mix of “soft” (behaviour change) measures and “hard” (infrastructure) measures; the reach of active transport initiatives; and the sustainability of change.

## Figures and Tables

**Table 1:** Active transport at pilot and comparison schools.

	Inner suburban		Outer suburban	
Year	Pilot	Comparison	Pilot	Comparison
	*n* (%)	*n* (%)	*n* (%)	*n* (%)
All active transport modes combined, observational counts				

2006	113 (31.4)	157 (40.8)	96 (18.9)	179 (24.6)
2007	140 (39.0)	142 (36.8)	167 (19.7)	231 (29.8)
Change	+7.6%	−4.0%	+0.8%	+5.2%
*P* value	**0.033**	0.256	0.717	**0.025**

Walking and cycling trips to school, grade 5 and 6 students, classroom Hands Up! surveys				

2006	131 (43.2)	206 (50.1)	104 (26.6)	164 (34.6)
2007	208 (52.0)	226 (45.8)	118 (25.0)	217 (31.2)
Change	+8.8%	−4.3%	−1.6%	−3.4%
*P* value	**<0.0002**	0.200	0.593	0.220

Number of bicycles observed in school grounds				

2006	8	6	26	N/A*
2007	14	7	22	11
Change	+6	+1	−4	N/A
*P* value	0.192	0.783	**0.015**	N/A

*Bike storage could not be located at this school at baseline.

Bold: *P* < 0.05.

**Table 2:** Parent characteristics at baseline and followup.

Demographic variable	Baseline		Followup		*P* value
	*n*	%	*n*	%	
Gender					
Female	365	89.0	315	89.0	0.920
Male	45	11.0	39	11.0	
Age					
18–29 years	11	2.7	3	0.8	
30–39 years	150	36.8	118	33.1	0.098
40–49 years	223	54.7	205	57.6	
50 years and over	24	5.8	30	8.4	
Country of birth					
Australia	347	84.6	299	83.8	0.823
Overseas	63	15.4	58	16.2	
Highest educational level achieved since leaving school					
No qualification	143	35.3	113	31.8	
Vocational qualification*	92	22.9	53	17.9	**0.042**
Diploma/associate diploma	48	11.7	46	12.8	
Bachelor/higher degree	112	27.2	117	34.1	

*Vocational qualification includes apprenticeship, trade certificate.

Bold: *P* < 0.05.

**Table 3:** Mode share of students' transport to and from school (% of trips), parent-reported data.

Travel mode	Baseline (*n* = 410)	Followup (*n* = 358)	*P* value
Car	46.3	44.2	0.076
Walk	27.8	28.7	0.352
Cycle	13.9	15.9	**0.015**
Scooter/skate	6.2	5.0	**0.026**
Other (public transport)	5.8	6.2	0.621
Total active trips (walk, cycle, and scooter/skate)	47.9	49.6	0.125

Bold: *P* < 0.05.

**Table 4:** Predictor variables for active transport to school, parent-reported baseline data.

Variable	Student use of active transport		*P*
	No active transport (no trips) n* (%)	Active transport (1–10 trips) n* (%)
School location			
Regional	112 (40.3)	166 (59.7)	**0.050**
Metropolitan	40 (30.3)	92 (69.7)	
Child gender			
Male	70 (37.0)	119 (63.0)	0.952
Female	81 (37.3)	136 (62.7)	
Child grade			
Grade 4	59 (48.8)	62 (51.2)	**0.007**
Grade 5	44 (32.6)	91 (67.4)	
Grade 6	48 (32.0)	102 (68.0)	
Distance to school			
Less than 0.5 km	3 (4.7)	61 (95.3)	
0.5–1 km	7 (8.5)	75 (91.5)	
1.1–2 km	30 (29.1)	73 (70.9)	**0.000**
2.1–4 km	33 (47.1)	37 (52.9)	
4.1–10 km	47 (82.5)	10 (17.5)	
More than 10 km	27 (100.0)	0 (0.0)	
Number of children in household			
One	20 (32.3)	42 (67.7)	
Two	68 (34.7)	128 (65.3)	0.405
Three	47 (43.1)	62 (56.9)	
Four or more	17 (39.5)	26 (60.5)	
Number of cars per adult in household**			
None	0 (0.0)	11 (100.0)	**0.001**
One	130 (36.0)	231 (64.0)	
Two	22 (59.5)	15 (40.5)	
Parent's country of birth			
Australia	130 (37.6)	216 (62.4)	0.521
Overseas	21 (33.3)	42 (66.7)	
Parent education			
No qualification since secondary school	58 (40.0)	87 (60.0)	0.364
Vocational qualification or higher	90 (35.4)	164 (64.6)	
Parent employment			
Both parents work full-time	43 (39.8)	65 (60.2)	0.492
At least one parent works part-time or is not employed	109 (36.1)	258 (62.9)	
How often parent cycles			
Once per month or less	126 (39.4)	194 (60.6)	0.069
At least once per week	26 (28.9)	64 (71.1)	
How often spouse/partner cycles			
No spouse or partner/once per month or less	133 (40.3)	197 (59.7)	**0.011**
At least once per week	19 (24.7)	58 (75.3)	
Walking to or from school on a regular basis is a possibility for child			
No	100 (83.3)	20 (16.7)	**0.000**
Yes/maybe	51 (17.7)	237 (82.3)	
Cycling to or from school on a regular basis is a possibility for child			
No	89 (64.0)	50 (36.0)	**0.000**
Yes/maybe	61 (23.1)	203 (76.9)	

*Number of students.

**0.5 cars were rounded up to one.

Bold: *P* < 0.05.

**Table 5:** Impact of the Ride2School program on students' use of active transport at least once per week, unadjusted and adjusted for predictor variables, parent-reported data.

Variable	OR	95% CI	*P*	AOR	95% CI	*P*
Data collection period (reference = baseline)	1.30	0.95–1.79	0.107	1.67	1.04–2.70	**0.035**
School location (reference = metropolitan)	0.64	0.41–1.00	**0.050**	1.12	0.77–1.63	0.559
Child grade				1.64	1.33–2.03	**0.000**
Grade 4	Reference					
Grade 5	1.97	1.19–3.27	**0.008**			
Grade 6	2.02	1.23–3.32	**0.005**			
Distance to school				0.42	0.35–0.51	**0.000**
Less than 0.5 km	8.36	2.43–28.72	**<0.000**			
0.5–1 km	4.4	1.82–10.65	**0.001**			
1.1–2 km	Reference					
2.1–4 km	0.46	0.24–0.87	**0.016**			
4.1–10 km	0.09	0.04–0.20	**<0.000**			
More than 10 km	Not applicable (zero in one cell)					
Number of cars per adult				0.48	0.14–1.65	0.245
None	Not applicable (zero in one cell)					
One	Reference					
Two	0.38	0.19–0.77	**0.005**			
How often spouse/partner cycles	0.49	0.28–0.85	**0.011**	1.79	0.68–4.71	0.239
Walking to school is a possibility	0.43	0.02–0.08	**0.000**	5.63	3.48–9.11	**0.000**
Cycling to school is a possibility	0.17	0.11–0.26	**0.000**	2.81	1.52–5.18	**0.001**

Bold: *P* < 0.05.

**Table 6:** Student characteristics at baseline and followup.

Demographic variable	Baseline		Followup		*P* value
	*n*	%	*n*	%	
Grade					
4	140	29.2	133	33.8	
5	166	34.7	138	35.0	0.228
6	173	36.1	123	31.2	
Gender					
Female	253	52.8	207	51.4	0.718
Male	226	47.2	196	48.6	
Age (years)					
Mean	10		11		**<0.0001**
Range	8–13		8–13		

Bold: *P* < 0.05.

**Table 7:** Mode share of students' transport to and from school (% of trips), student reported data.

Travel mode	Baseline *n* = 479	Followup *n* = 403	*P* value
Car	42.9	43.1	0.920
Walking	28.4	27.7	0.464
Cycling	15.5	16.1	0.450
Scooter/skate	7.2	4.9	<**0.0002**
Other (public transport)	6.0	8.2	<**0.0002**
Total active trips (walk, cycle, and scooter/skate)	51.1	48.7	**0.029**

Bold: *P* < 0.05.

**Table 8:** Predictor variables for active transport to school, student baseline data.

Variable	Student use of active transport		*P*
	No active transport (no trips) n* (%)	Active transport (1–10 trips) n* (%)	
School location			
Regional	110 (31.8)	236 (68.2)	**0.043**
Metropolitan	29 (22.3)	101 (77.7)	
Child gender			
Male	62 (27.6)	163 (72.4)	0.455
Female	77 (30.7)	174 (69.3)	
Child grade			
Grade 4	48 (34.8)	90 (65.2)	
Grade 5	48 (29.1)	117 (70.9)	0.160
Grade 6	43 (24.9)	130 (75.1)	
Whether student cycles after school hours			
Never/sometimes	71 (33.2)	143 (66.8)	0.077
Quite often/most days	67 (25.8)	193 (74.2)	
Whether adults in the household cycle			
No	58 (33.5)	115 (66.5)	0.114
Yes	80 (26.7)	220 (73.3)	
Whether student had participated in Bike Ed.			
No	59 (26.1)	167 (73.9)	0.158
Yes	80 (32.0)	170 (68.0)	

*Number of students.

Bold: *P* < 0.05.
